# Deep learning-based classification of coronary arteries and left ventricle using multimodal data for autonomous protocol selection or adjustment in angiography

**DOI:** 10.1038/s41598-025-99651-z

**Published:** 2025-04-30

**Authors:** Arpitha Ravi, Philipp Bernhardt, Mathis Hoffmann, Florian Kordon, Siming Bayer, Stephan Achenbach, Andreas Maier

**Affiliations:** 1 Pattern Recognition Lab, Department of Computer Science, Friedrich-Alexander-University Erlangen-Nürnberg (FAU), 91058 Erlangen, Germany; 2 Siemens Healthineers AG, 91301 Forchheim, Germany; 3 Department of Medicine 2-Cardiology and Angiology, Friedrich-Alexander-University Erlangen-Nürnberg (FAU), Universitätsklinikum, Erlangen, 91054 Germany

**Keywords:** Cardiac anatomy classification, Deep learning, X-ray imaging parameter optimization, Biomedical engineering, Interventional cardiology

## Abstract

Optimal selection of X-ray imaging parameters is crucial in coronary angiography and structural cardiac procedures to ensure optimal image quality and minimize radiation exposure. These anatomydependent parameters are organized into customizable organ programs, but manual selection of the programs increases workload and complexity. Our research introduces a deep learning algorithm that autonomously detects three target anatomies:the left coronary artery (LCA), right coronary artery (RCA), and left ventricle (LV),based on singleX-ray frames without vessel structure and enables adjustment of imaging parameters by choosing the appropriate organ program. We compared three deep-learning architectures: ResNet-50 for image data, a Multilayer Perceptron (MLP) for angulation data, and a multimodal approach combining both. The dataset for training and validation included 275 radiographic sequences from clinical examinations, incorporating coronary angiography, left ventriculography, and corresponding C-arm angulation, using only the first non-contrast frame of the sequence for the possibility of adapting the system before the actual contrast injection. The dataset was acquired from multiple sites, ensuring variation in acquisition and patient statistics. An independent test set of 146 sequences was used for evaluation. The multimodal model outperformed the others, achieving an average F1 score of 0.82 and an AUC of 0.87, matching expert evaluations. The model effectively classified cardiac anatomies based on pre-contrast angiographic frames without visible coronary or ventricular structures. The proposed deep learning model accurately predicts cardiac anatomy for cine acquisitions, enabling the potential for quick and automatic selection of imaging parameters to optimize image quality and reduce radiation exposure. This model has the potential to streamline clinical workflows, improve diagnostic accuracy, and enhance safety for both patients and operators.

## Introduction

### Optimizing imaging for cardiac procedures: challenges and innovations in interventional cardiology

In the year 2020, European Society of Cardiology (ESC) member countries reported a median of 4084 diagnostic coronary catheterization procedures and 1879 percutaneous coronary interventions per one million inhabitants^1^. Invasive, catheter-based 2D X-ray imaging provides real-time visualizations of the coronary arteries and other cardiac structures. To achieve optimal image quality at minimal radiation exposure, precise parameterization of acquisition protocols is necessary^2–4^, and factors such as the target anatomy, the degree of expected motion, and the amount of contrast that will be present in the image must be considered. Hence, specialized programs tailored to specific imaging tasks have been developed^5,6^. In clinical practice, they are often referred to as “organ programs”. In the context of coronary angiography, coronary intervention, and cardiac structural procedures such as transcatheter aortic valve implantation, operators presently select the imaging parameters manually. Commercial imaging systems offer organ-specific programs, and the operators choose default parameters for the entire diagnostic procedure, with adjustments made seldomly. This can lead to oversight and missed opportunities to optimize image quality and minimize radiation exposure. By adjusting parameters based on target anatomy and physiological factors, these risks can be avoided.

**Fig. 1:**
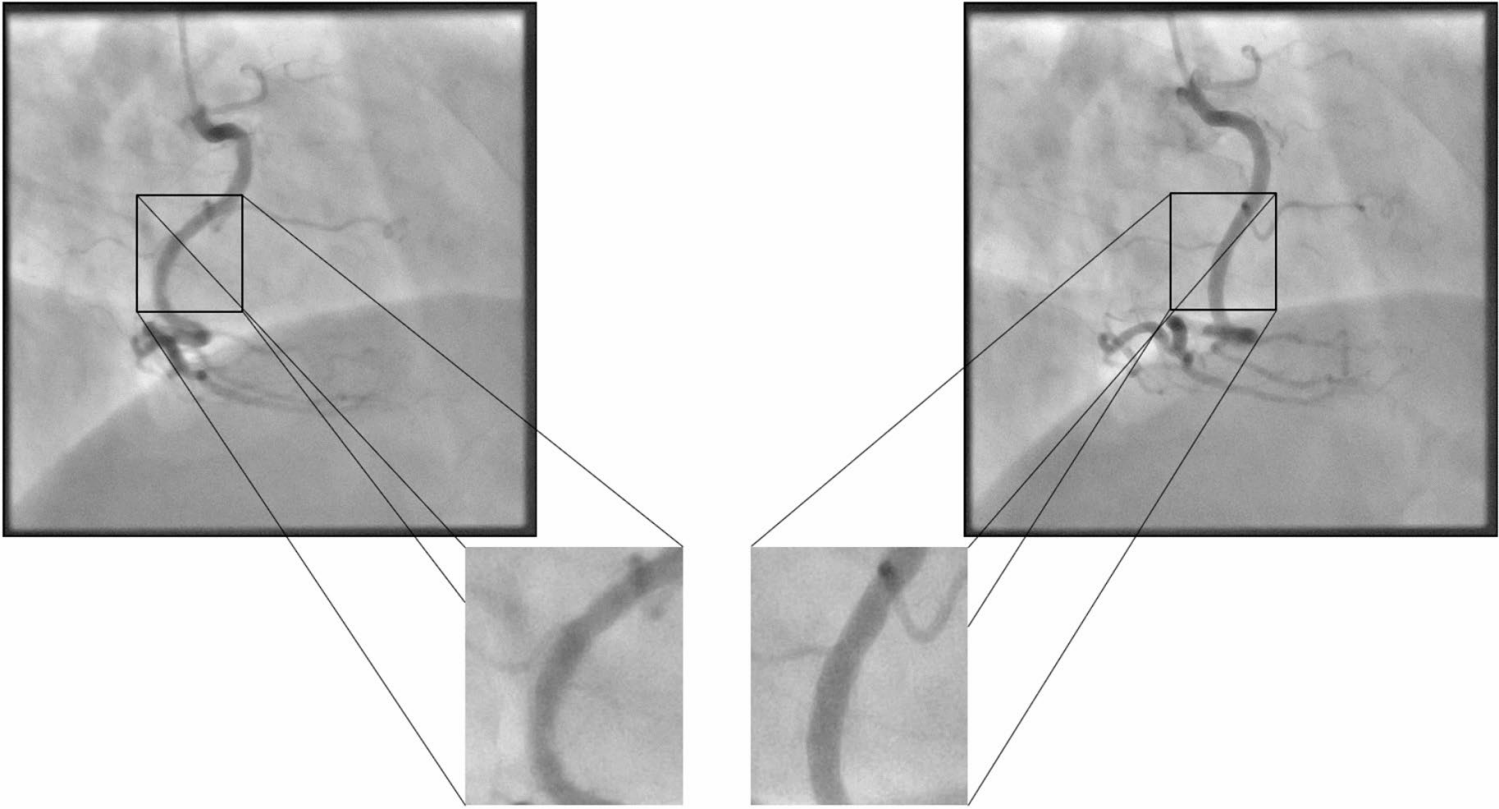
Typical coronary angiography acquisitions. (left), right coronary artery (RCA) displays blurry vessels due to motion blur, which is because of non-optimal parametrization (right), RCA that has been parameterized correctly shows a clear vessel structure.

Figure 1 represents the addressed problem. It presents radiographs showing the right coronary artery (RCA). As we notice, the left image displays a blurry and shaken vessel structure as a result of motion blur. This is because the parameterization was not optimal. The exposure time is optimized as per the left coronary artery (LCA) to get the highest possible contrast, but the RCA requires a lower exposure time due to its higher velocity, resulting in blurry vessels. The blurriness is towards the direction of motion. However, in the right image, we notice the clear vessel structure because the exposure time is correctly balanced according to the velocity of the RCA.

Efficient workflow is critical in cardiology, and an autonomous system capable of recognizing the coronary segment and adjusting imaging protocols may enhance diagnostic procedures and percutaneous coronary intervention (PCI). By selecting suitable organ programs or advising the operator about the same, this innovation could reduce manual steps and radiation exposure while ensuring optimal image quality for safer diagnostic and interventional procedures.

### Automated coronary imaging program selection with deep learning

This paper proposes deep-learning algorithms to automate the detection of the target anatomy and the selection of an appropriate organ program in invasive 2D X-ray imaging. Our study evaluates the effectiveness of this approach for identifying and classifying three cardiac structures: the left coronary artery (LCA), right coronary artery (RCA), and left ventricle (LV) since the imaging of these anatomical structures could allow operators to have dedicated imaging protocols, overcoming the limitations of generic programs. For continuous adaptation and optimization of image acquisition parameters, it would be extremely important to do so rapidly, before the diagnostically relevant frames are acquired. Therefore, identifying the imaged anatomy before injecting the contrast medium is essential, typically during the fluoroscopy stage that precedes cine angiographic acquisitions. Alterations to the imaging protocol during the acquisition of a cine angiographic scene could lead to distractions for the observer and possibly compromise diagnostic integrity. Hence, we use images without arterial information, except for the catheter tip. This makes the detection and classification process more challenging. Furthermore, the dataset also include images that are sometimes zoomed in, making the anatomy detection difficult.

Additionally, we explore using C-arm angulation for classification tasks and develop a multimodal approach. This approach uses image and angulation data to accurately identify the given anatomy.

### Key contributions

We developed and tested three deep-learning methods for target anatomy classification using image and C-arm angulation data. Our key contributions are as follows:

- The research involves evaluating various image pre-processing techniques and angulation data for classifying LCA, RCA, and LV.
- Introduced a novel multimodal approach combining angulation with image data, improving classification accuracy.
- Conducted an inter-rater study comparing our model’s performance to experts, validating its practical value.

## Theoretical background

### Image pre-processing

**Fig. 2:**
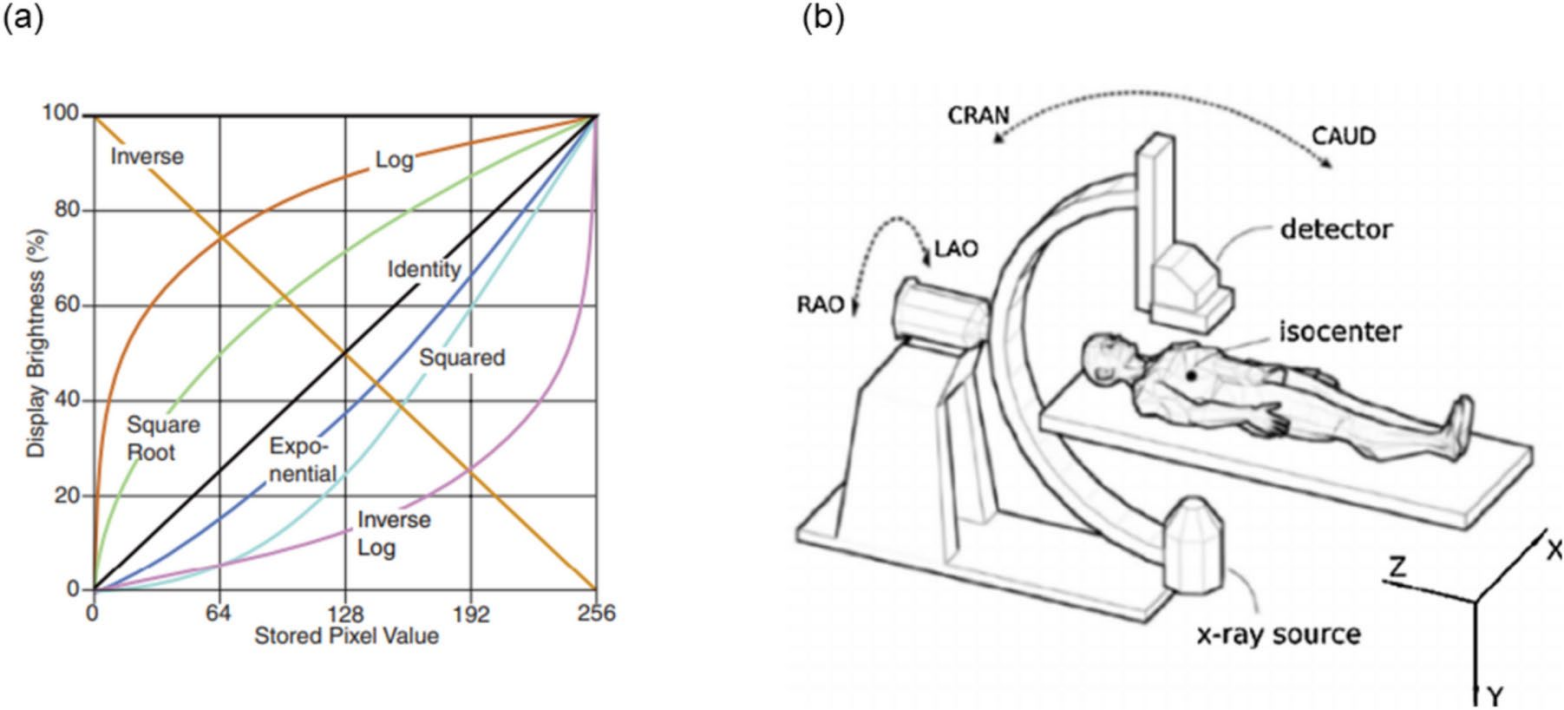
Illustration of different pixel representations and the X-ray acquisition setup with standard terminology for C-arm angulation. (**a**) Plot depicting the effects of the square root and log transformation on the displayed brightness^8^. (**b**) Orientation angles of the C-arm^9^.

In machine learning, data representation is crucial, especially for tasks like classification, regression, detection, or segmentation, particularly with image data. Obtaining a meaningful pixel representation is essential for effective learning^7^. However, different image pre-processing techniques have varying effects on feature extraction, which affects task outcomes. While some techniques enhance image quality, others may lead to excessive manipulation, resulting in suboptimal results. Figure 2a illustrates the effects on displayed brightness. Thus, we evaluate various image pre-processing techniques to assess their impact on our classification task. The following are the different image pre-processing techniques that were evaluated.

- Log transform: Logarithm operation is a non-linear transformation that enhances contrast^10,11^. The log of an image compresses the displayed brightness at the bright end and expands the dark end. Ultimately, all contrasts are harmonized in the whole image without dependency on the background.
- Square root transform: Squareroot is also a non-linear transformation that evens out pixel intensities. The square root focuses on harmonizing the noise in the whole image, which originally follows the counting statistics in the raw image^12^. This helps when performing a filtering task.
- Scatter correction and log: It is a technique used to remove the effects of scattered radiation on the original image signal. For the scatter correction, an image is read, and a scatter offset is subtracted from it. To obtain this offset, we first consider a portion of the collimator in the image. The mean value of this collimator region is calculated, which is the offset. It is then subtracted from the original image. Next, the log transformation is applied to the corrected image.

Additionally, our objective for exploring this information was to utilize the concept of known operator learning^13^, to evaluate its potential advantages when incorporated into our classification task using deep learning. The above image processing techniques are used in the imaging pipeline of our system. Hence, we chose them for our experiment to evaluate its effect on the final classification task. After applying the aforementioned techniques, the images are normalized using the z-score normalization. The resulting images are then used as input for network training. The image only with z-score normalization is used as a baseline for comparison.

### C-arm angulation data

In our study, we want to explore the use of multimodal learning^14–16^ for anatomy classification. We extend beyond the utilization of image datasets by integrating the C-arm angulation data which is a key factor in medical diagnostic imaging. Angulations are systematically categorized as LAO (Left Anterior Oblique) and RAO (Right Anterior Oblique) for lateral views, and CAUD (Caudal) and CRAN (Cranial)^17^ for vertical orientations. Figure 2b provides a graphical representation of these orientations. Our analysis incorporates the CRAN and RAO angles derived from the C-arm system.

### Literature

Current studies on coronary arteries are focused on methods that use the vessel structure mainly for pathology detection. Deep learning techniques have been used for segmentation and classification of the right and left coronary arteries for stenosis detection^18–21^. In this project, however, we target anatomy detection without using vessel structures, specifically to support organ program selection from the first few frames of an acquisition, making our task and approach novel.

## Results

Since the distribution of our test data is slightly imbalanced, we use the F1-score, AUC, and confusion matrix for an extensive evaluation of our deep learning models. We present an ablation study by separately evaluating image and angulation datasets for the classification task, followed by an assessment of our multimodal model that integrates both data sources.

### Comprehensive model assessment and experimental insights

Before conducting a detailed analysis of the image and angulation data, we performed preliminary experiment, as presented below. Given that the task involves image classification, we utilized a ResNet-50 model. Two versions of the model were trained: a pre-trained ResNet-50 and a ResNet-50 trained from scratch.

**Table 1:** Evaluation scores obtained from training Resnet-50 model from scratch and using a pre-trained model.

	Resnet-50 from scratch		Pre-trained Resnet-50	
Class	F1-score	AUC	F1-score	AUC
LCA	0.54	0.65	0.63	0.75
RCA	0.49	0.65	0.61	0.72
LV	0.68	0.78	0.73	0.82

Table 1 presents the results of both Resnet-50 trained from scratch and pre-trained model. We observe that the pre-trained model outperforms the model trained from scratch, achieving a macro F1-score of 0.66 and an average AUC of 0.76, compared to a macro F1-score of 0.57 and an average AUC of 0.69 for the latter. This performance gap is likely due to using a limited dataset on a complex model, making transfer learning a more effective approach by utilizing pre-trained features and mitigating overfitting^22^. Therefore, the pre-trained ResNet-50 model was selected for further analysis.

#### Classification using X-ray image data

**Fig. 3:**
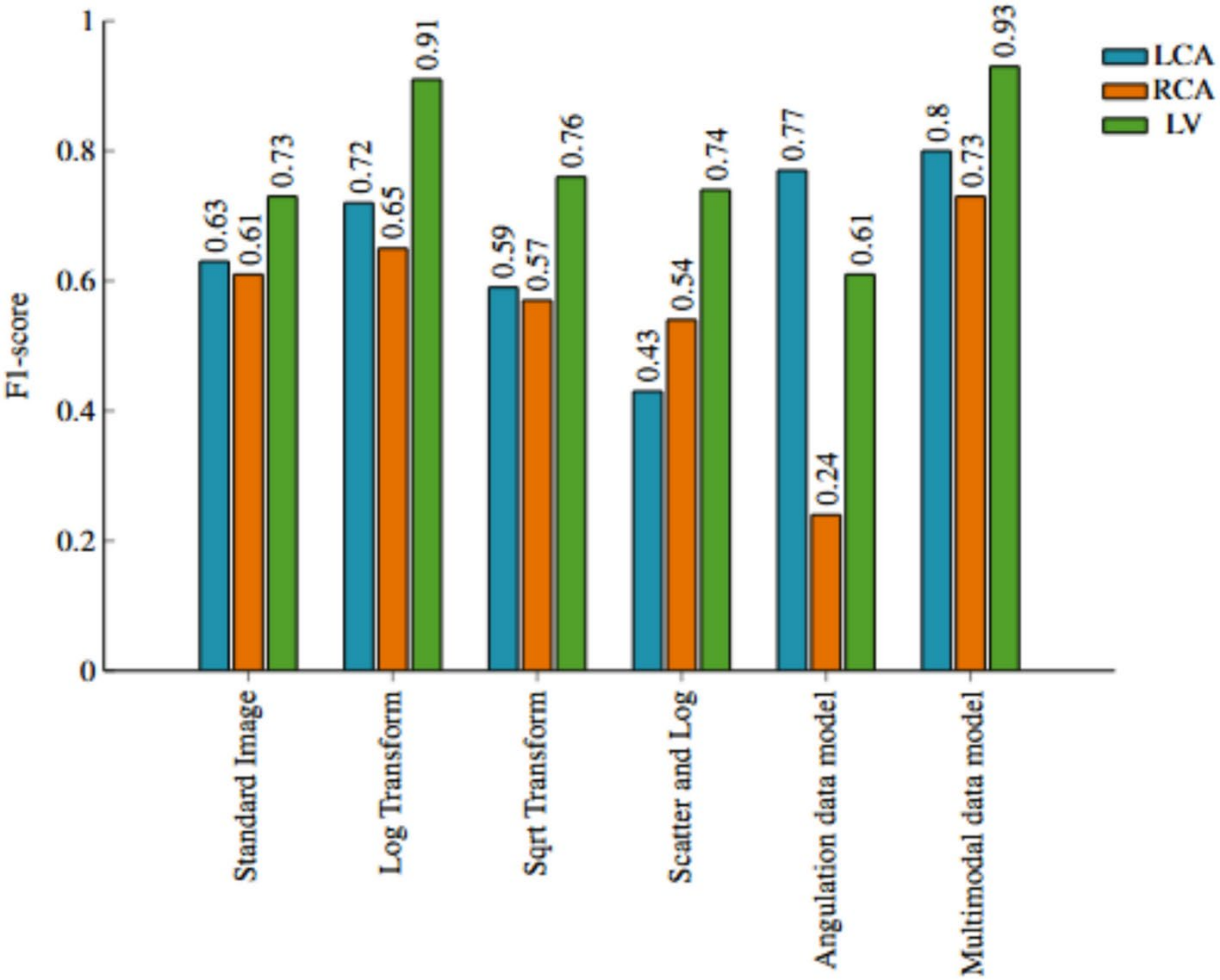
Comparison of class-wise F1 scores for the image data model across different preprocessing techniques, alongside results from the angulation data and multimodal data models.

**Fig. 4:**
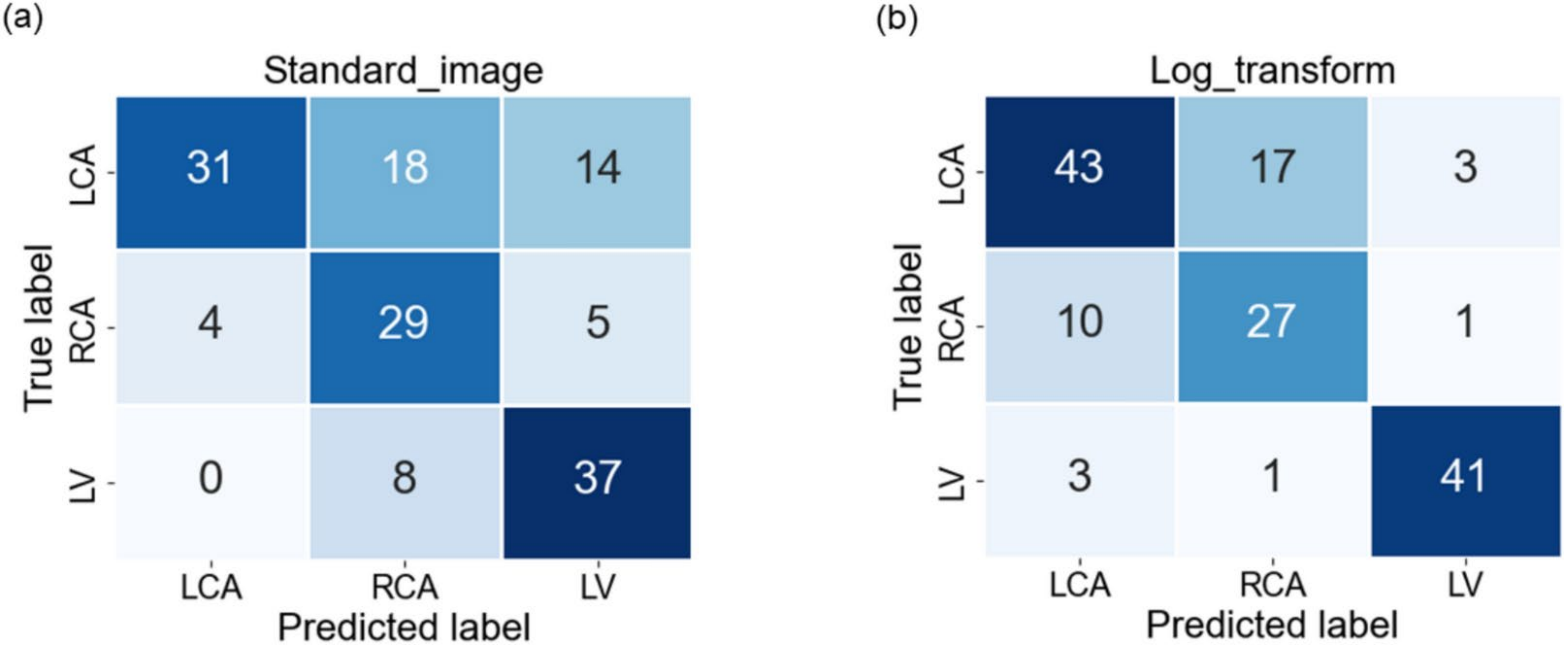
Confusion Matrices obtained from the model evaluation on (**a**) Standard image and (**b**) Log transformed image.

**Fig. 5:**
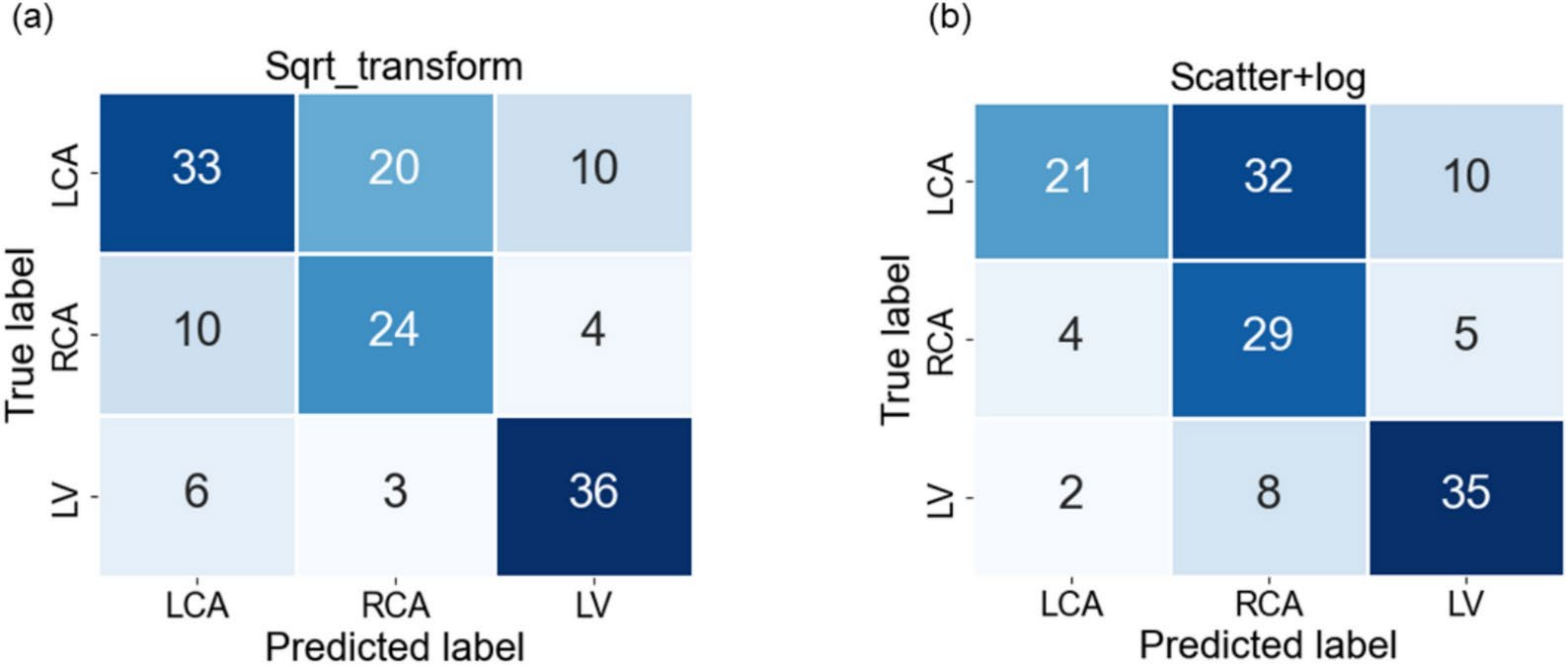
Confusion Matrices obtained from the model evaluation on (**a**) Square-root image and (**b**) Scatter corrected Log transformed image.

In Fig. 3, we observe the classwise evaluation obtained using different image pre-processing techniques on test data using the pretrained Resnet-50 model. The log-transformed images consistently outperform other methods for all three classes with F1-scores of 0.72, 0.65, and 0.91, and AUC of 0.76, 0.77, and 0.94 for LCA, RCA, and LV respectively. The scatter correction method with log transformation results in the lowest F1-scores of 0.43, 0.54, and 0.74 and AUC of 0.61, 0.70 and 0.81 for LCA, RCA, and LV respectively. Scatter correction manipulates images by subtracting an offset, and may adversely affect the model’s performance. Although scatter-corrected images may appear best for the human eye, they may not be the best for the network. The log transformation can enhance the visibility of darker regions while compressing brighter regions. This visibility enhancement could allow the network to extract more valuable information from the images, ultimately improving classification performance. However, model performance in the LCA and RCA classes is lower compared to that in the LV class. The confusion matrices in Figs. 4 and 5, reveal that LCA and RCA are often misclassified. These misclassifications could be due to common features between the two classes, contributing to suboptimal scores in our observations. This can also be observed in the AUC values. Another noteworthy finding is the excellent performance of log-transformed images on LV class with an F1-score of 0.91. This could be attributed to the improved visibility offered by log images, as previously discussed. These findings highlight using log-transformed images to enhance the model’s performance.

#### Classification using C-arm angulation data

**Fig. 6:**
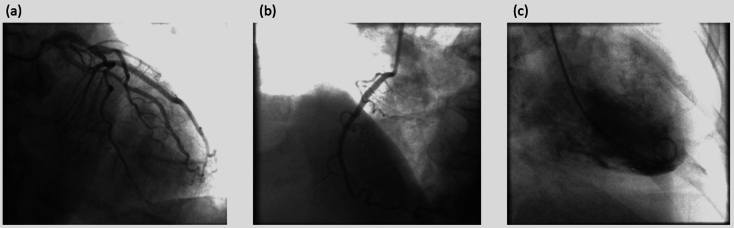
Angiographic frame after injection of contrast agent. (**a**) Angiogram of the left coronary artery (**b**) Angiogram of right coronary artery (**c**) Angiogram of the left ventricle.

Table 2 presents the results derived from the model when applied to the test data. Notably, the model’s performance excels in the LCA class with an F1 score of 0.77 and AUC of 0.79 compared to the other two classes. The reason for this is clear when observing Fig. 6, which shows a plot of all the angulation data points in the distribution. Upon closer analysis of the class distribution, it becomes apparent that these classes are not linearly separable due to the overlap between them. Specifically, the RCA and LV classes exhibit notable overlap, rendering them indistinguishable, whereas the LCA class stands out as distinguishable. The implications of this distribution are further reflected in the performance metrics, with the RCA class demonstrating the lowest scores with an F1-score of 0.24 and 0.55 AUC. The model exhibits a notably low capability in correctly identifying all images belonging to the RCA class, highlighting challenges in accurately recognizing cases within this category. A deeper understanding of this issue can be obtained by reviewing the associated confusion matrix.

**Table 2:** Evaluation scores obtained using the angulation data model on test dataset.

Class	F1-score	AUC
LCA	0.77	0.79
RCA	0.24	0.55
LV	0.61	0.72

**Fig. 7:**
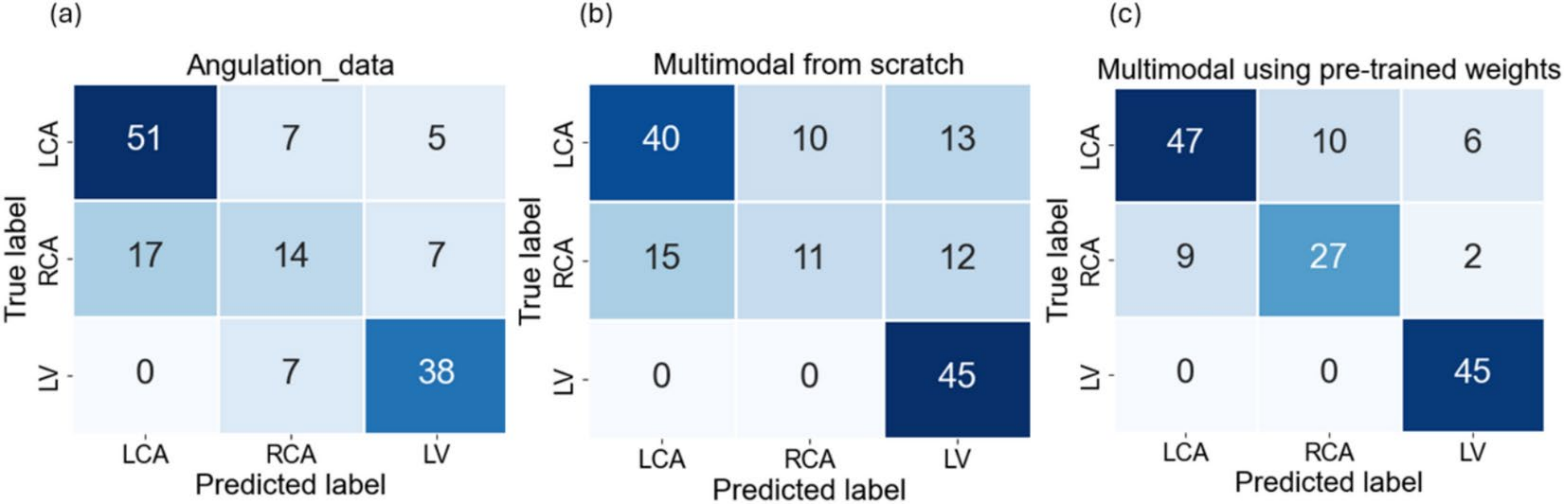
Confusion Matrices obtained from the model evaluation on (**a**) Angulation data and (**b**) Multimodal data model trained from scratch (**c**), Multimodal data model trained with pre-trained weights.

Figure 7a presents the confusion matrix of the angulation data model. It is evident from this matrix that the model correctly predicts the majority of LCA samples i.e., 51 out of 63. In contrast, only a few RCA samples i.e., 14 out of 38 are accurately classified. This discrepancy can be because of the misclassification of RCA samples as LCA and LV,  which is directly linked to the aforementioned issues of class overlap. Similarly, misclassified LV samples are often incorrectly categorized as RCA. In summary, the angulation data model effectively classifies the LCA class which could be because it has more samples compared to the other two. However, it performs less optimally for RCA and LV classification.

#### Classification using multimodal data

The results obtained using the multimodal data are presented in this section. Table 3 presents the performance comparison of a ResNet-50 and MLP-based model trained from scratch versus one utilizing pre-trained weights with frozen layers. Training the model from scratch using the default ResNet-50 hyperparameters yielded suboptimal results due to discrepancies between the hyperparameter configurations of ResNet-50 and MLP which is observed in table 3. Therefore, a fine-tuning approach was adopted, where only the final fully connected layers were trainable. Since, the log-transformation model exhibited the highest performance while using image data, and its weights were utilized for fine-tuning, along with those from the angulation data model. Notably, the model using pre-trained weights demonstrated superior performance. The model trained from scratch achieved a macro F1-score of 0.65, whereas the pre-trained model attained a macro F1-score of 0.82. Both models correctly classified all samples of LV class correctly as observed in the confusion matrices in Fig. 7b,c. This could be attributed to the strong performance of the log-transformation model on image data. However, the LCA and RCA classes exhibited lower accuracy, particularly in the model trained from scratch, which struggled with misclassifications, especially in the RCA class. The pre-trained model demonstrated improved performance, achieving F1-scores of 0.8 and 0.73, with AUC values of 0.83 and 0.81 for LCA and RCA, respectively. Despite this improvement, these two classes still had lower scores compared to LV, primarliy due to misclassifications between LCA and RCA, similar to the model trained from scratch. Overall, integrating multimodal data into the pre-trained model enhanced network performance compared to models relying solely on image or angulation data. This suggests that combining image and angulation information effectively improves classification accuracy while fine-tuning the model using pre-trained weights.

**Table 3:** Evaluation scores obtained using the multimodal data model on the test dataset.

Class	Multimodal model trained from scratch		Pre-trained Multimodal model	
	F1-score	AUC	F1-score	AUC
LCA	0.70	0.75	0.80	0.83
RCA	0.37	0.60	0.73	0.81
LV	0.88	0.94	0.93	0.97

#### Comparison of models

**Fig. 8:**
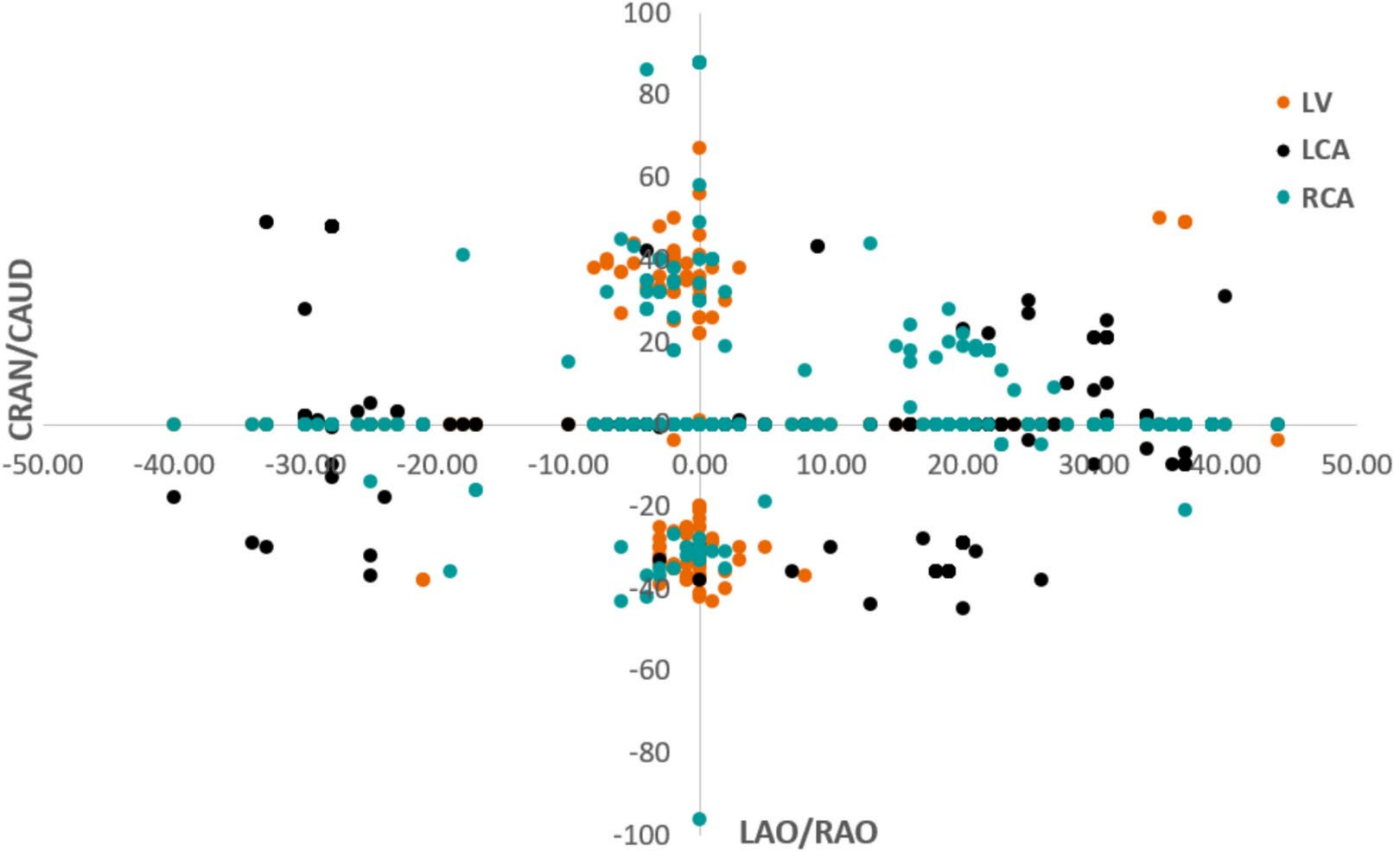
Plot depicting the distribution of the angulation data for all three class labels LCA, RCA, and LV.

**Fig. 9:**
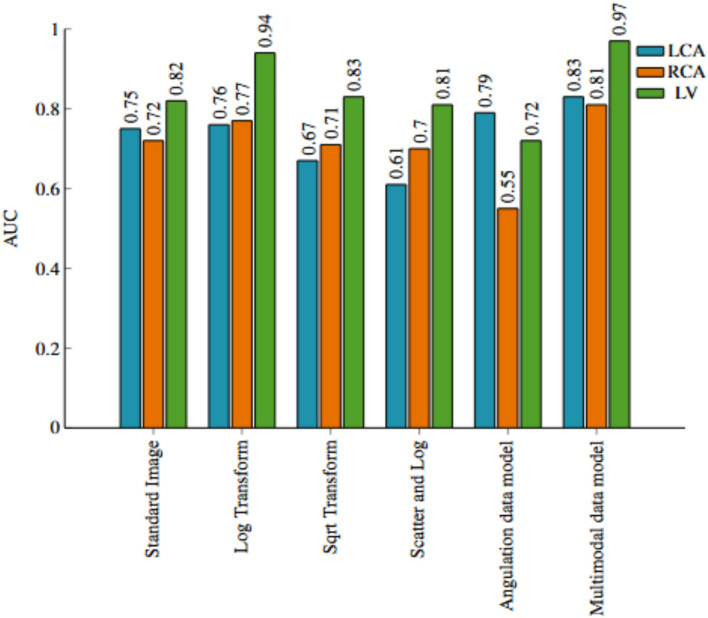
Comparison of class-wise AUC for the image data model across different preprocessing techniques, alongside results from the angulation data and multimodal data models.

Figures 3 and 6 illustrate the class-specific F1 scores and AUC values across all models. For a more effective comparison, we reference the results of the log-transformed image data model, which outperformed other pre-processing techniques on the test dataset. Likewise, for the multimodal approach, we present the model utilizing pre-trained weights, as it achieved the highest performance. Based on our evaluation, we have observed that combining X-ray images and angulation data improves model performance. The model based solely on angulation data had the lowest scores, which could be attributed to the overlap of classes. As seen in Fig. 8, the overlap between the classes might have decreased the model’s performance. After analyzing the confusion matrices of all the methods, we observed that the model based on angulation data was the most successful in accurately identifying the maximum number of LCA classes as compared to other models. The model could identify 51 samples correctly out of 63 as seen in Fig. 7a. However, it was the weakest model for the RCA and LV class. Similarly, the image data model (log transformation) had some drawbacks, especially with the LCA and RCA class labels. From Fig. 4b it can be noticed that 17 LCA images of 63 are misclassified as RCA and 10 RCA images of 38 are misclassified as LCA, which could be attributed to the similarity in features between the two classes. To analyze the significance, we performed McNemar’s test and concluded that using image data for predicting the anatomy performed significantly better than using angulation data (p< 0.001). Further, we found that combining image and angulation data in a multimodal model slightly improves the classification performance, compared to using only image data, which can be observed in Figs. 3 and 9. The model resulted in F1-scores of 0.8, 0.73, and 0.93 and AUC of 0.83, 0.81, and 0.97 for LCA, RCA, and LV classes respectively.

### Inter-rater study

**Fig. 10:**
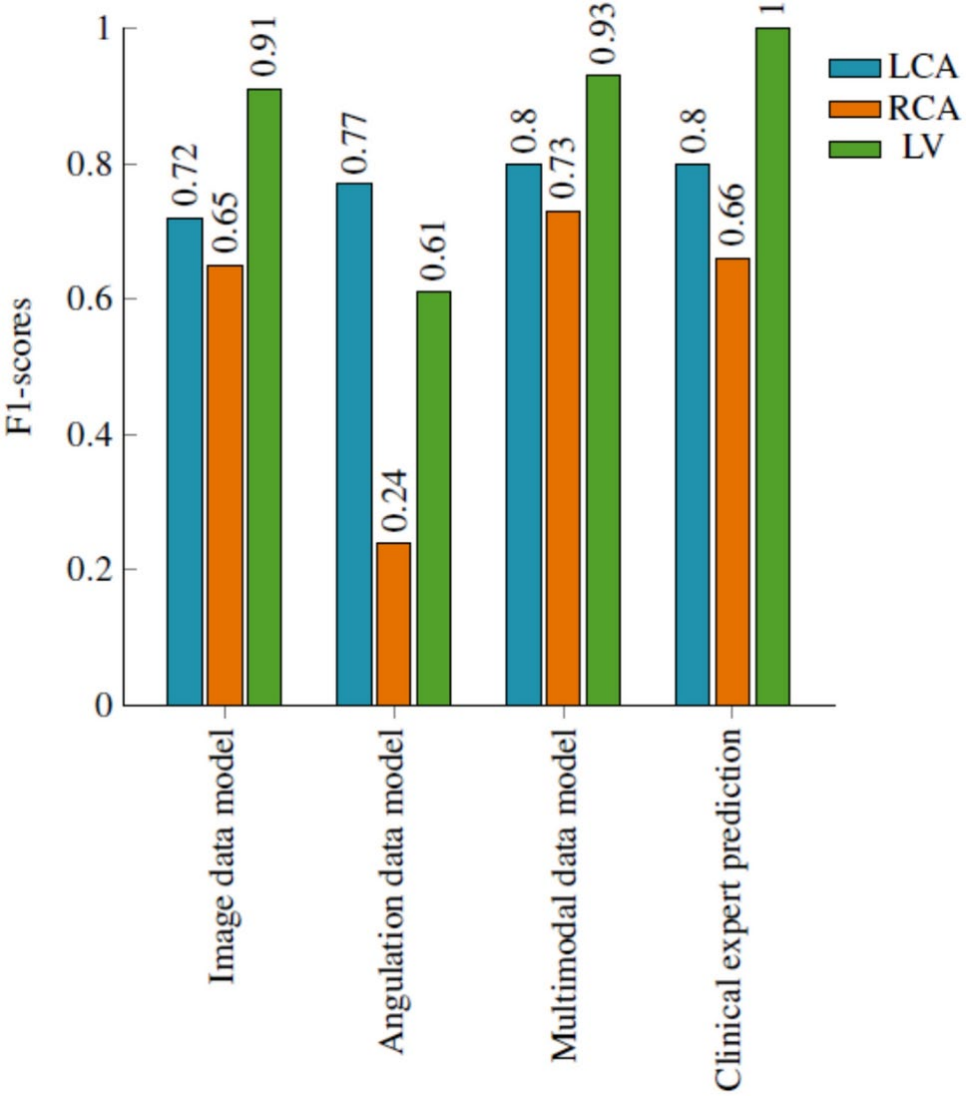
Class-wise F1-score presenting a comparison between the image data model, angulation data model, multimodal data model, and clinical expert prediction.

In Fig. 10 we present a plot that illustrates the comparison between our deep learning models and the average predictions made by clinical experts. Notably, our multimodal model’s performance closely aligns with the evaluations provided by these experts with average F1-scores of 0.82, particularly in the LCA and RCA anatomical regions, demonstrating notable accuracy. This similarity in performance is attributed partly to the inherent challenge of identifying anatomy without specific information related to the artery. It’s worth highlighting that the LV (Left Ventricle) class attains the highest scores with an F1-score of 1.0, in terms of expert predictions. This is primarily because LV images typically feature pigtail catheters, which experts can readily identify without relying on the use of contrast medium, as depicted in Fig. 7b. Based on our comprehensive evaluation, it can be concluded that our model performs distinctively, and yields results that are comparable to those provided by clinical experts in their assessments. Another key observation is that experts took an average of 3 to 4 minutes per image to identify the anatomy, unlike our model which has an inference time of 1–2 seconds. If the experts were given the same time frame as our model for evaluation, the results might differ.

## Discussion

We developed a deep-learning algorithm that automatically identifies the target anatomy: left coronary artery, right coronary artery, and left ventricle, based on non-contrast single x-ray frames and the corresponding angulation at the beginning of a cine angiographic sequence. The best results were obtained using a multimodal approach combining image data with information on C-arm angulation. The pre-trained Resnet-50 model showed that using log transformation as a pre-processing step produced the best results compared to other methods. The log-transformed images produced a macro F1-score of 0.76 and an average AUC of 0.82. However, the angulation data model did not yield the best results due to misclassification. Further, combining image and angulation information resulted in better classification results for each class compared to the other two models. The multimodal model yielded a macro F1-score of 0.82 and an AUC of 0.87. Finally, our Inter-rater study proved that the multimodal model performance is similar to the expert performance with an average F1-score of 0.82 for both. This shows that our deep learning model can generalize on the cardiac anatomies, matching clinical experts suggesting further clinical studies.

## Limitations

Our deep learning model demonstrates comparable performance to clinical experts in distinguishing between the left coronary artery (LCA), right coronary artery (RCA), and left ventricle (LV). However, it cannot currently extract catheter position information, which is important for aiding in anatomical classification, particularly in left ventricle (LV) assessment. We employ a dataset comprising 275 training images and 146 test images. The algorithm could be more robust with improved scores by expanding this dataset to include a broader range of samples that include different acquisition parameters and patient statistics. While we have evaluated the model’s performance on images, we have yet to assess its impact on clinical workflow. Further evaluation is necessary to understand its implications during actual clinical use.

## Conclusion

Our proposed algorithm has demonstrated successful and reliable classification between LCA, RCA, and LV anatomies. The evaluation of various image pre-processing methods revealed that the log transformation technique yielded the most favorable classification outcomes, leading to its incorporation into the multimodal data model. The multimodal data model demonstrated improved F1-scores and AUC for individual classes and overall compared to both the image data model and the angulation data model. Currently, we are using ResNet-50 and MLP network architectures. In future work, we plan to explore additional architectures, such as transformers, and experiment with unsupervised methods to further improve performance. However, our current results justify that the classification task is possible without using vessel structure and further clinical evaluation of the model. We aim to expand the sample size and incorporate less commonly imaged pathologies, including cases like coronary artery bypass grafts. We plan to develop a prototype to test the impact of the algorithm on the clinical workflow. Additionally, we want to evaluate the effect of patient parameters, such as body weight and heart rate, on the anatomy classification.

## Methods

**Fig. 11:**
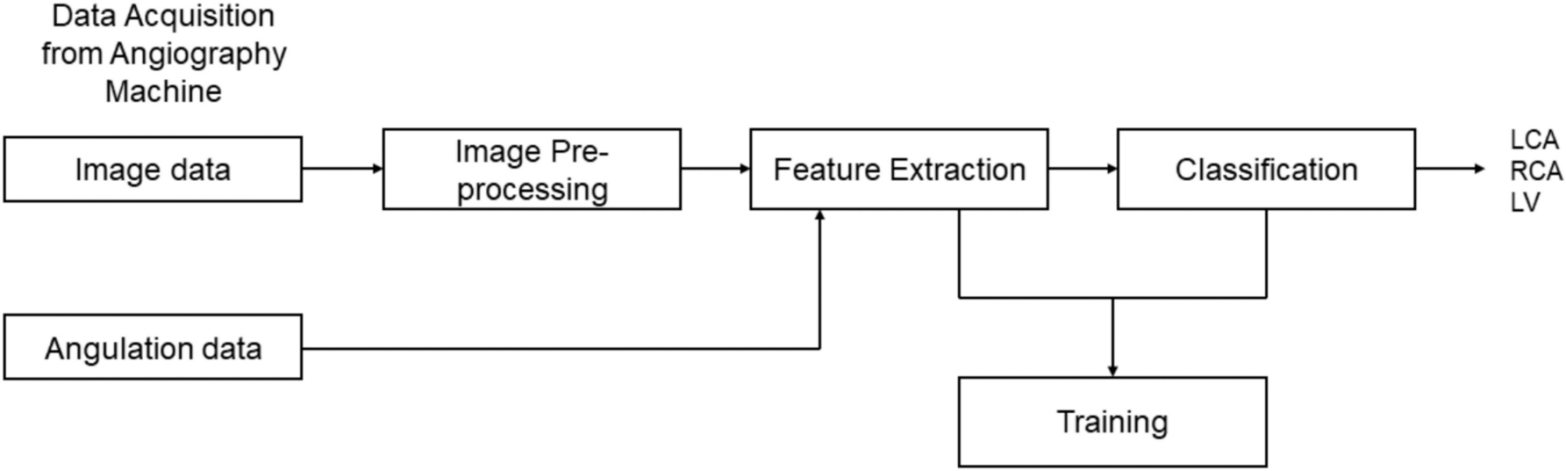
Overview of the methodology.

Figure 11 illustrates the methodology utilized in our paper. The input data, comprising image data and c-arm angulation information, is obtained from the X-ray angiography machine. The image data undergoes pre-processing before being input into the feature extractor, while the angulation data is directly fed to the feature extractor without pre-processing. The extracted features are then input into the classifier, which determines whether the input corresponds to the LCA, RCA, or LV class.

### Data overview and preparation

In this section, we detail the data utilized in this project, covering data collection, pre-processing, and its application in the testing, training, and validation phases.

#### Data characteristics

The dataset for this task comprises radiographic sequences from fluoroscopy (non-enhanced) and cine angiograms of left ventriculography and coronary angiography. To align with the project’s objective of real-time anatomy detection, the sequences are used in raw format for training purposes. Raw data in this context refers to the information directly obtained from the X-ray angiography system without undergoing pre-processing or filtering. The images are only corrected for detector artifacts, and no extra manipulation or adjustments are applied. Additionally, angulation data, providing C-arm positions during acquisitions with CRAN and RAO angles in degrees, is obtained from the stored image parameters.

To accommodate diverse imaging parameters, we utilize multisite data from various locations and multiple X-ray angiography systems (all manufactured by Siemens Healthineers). The dataset covers a period from 2010 to 2021. The images exhibit varying resolutions, ranging from a minimum of 896x896 to a maximum of 1920x2480. Since we want to use the frames without the vessel structure for training, we only consider the first frame of the sequence as it does not contain the artery information from using the contrast medium.

**Fig. 12:**
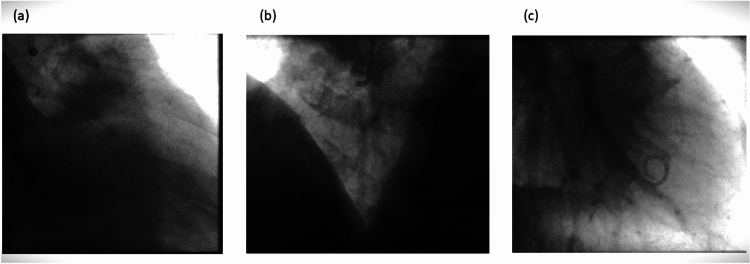
Angiographic frame just before the injection of contrast agent. (**a**) Angiogram of the left coronary artery (**b**) Angiogram of right coronary artery (**c**) Angiogram of the left ventricle.

The images in Fig. 12 illustrate the key anatomical regions relevant for the classification task-specifically, (a) the left coronary artery (LCA), (b) right coronary artery (RCA), and (c) left ventricle (LV), respectively-without displaying any arterial details. These pre-contrast injection images are used because the objective is to enable the system to identify the anatomy prior to the contrast-injection phase. In contrast Fig. 6 illustrates the contrast injection phase, where the visibility of the arterial structures simplifies the identification of anatomical regions. However, without the arterial information, the anatomies appear very similar, particularly in cases where images are captured at higher zoom levels making the classification task challenging.

However, in case of LV identification the pigtail catheter is a potential indicator unique to the procedure which makes identification of the anatomy much easier. The catheter’s position could also be utilized for RCA and LCA, i.e., the catheter would be pointing to the left side for RCA and right side in case of LCA, however, it’s essential to note that this information may not always be reliable because there are cases where the catheter is straight or pointing otherwise.

#### Data preparation

The raw images may have collimator information and flip or rotation errors, which we address by excluding the collimator area and correcting for flipping or rotation using stored parameters. Subsequently, we crop the images to eliminate the collimator area ensuring accurate classification without background noise interference. Since the images have variable resolution, we pad the images to match the highest resolution in the mini-batch during training. This choice of padding over resizing is deliberate, aiming to enable the network to learn from the entire anatomy without compromising anatomical details, which resizing might alter. Additionally, we employ two augmentation techniques to increase the training sample size, namely Gaussian noise, and blurring, to enhance the robustness of the training process. These two techniques were specifically chosen as they are common issues that could occur during imaging.

#### Annotation and model focus

The image data was annotated by clinical experts using the specialized EXACT annotation tool^23^. The clinical experts included image quality specialists, radiological technologists, and medical physicists with 6 to 10 years of experience in the field. Four clinical experts supported the task of annotation. The specialists assessed the entire sequence, especially during contrast injection, to gain insights into vessel structure. The sequences are input to the annotation tool, and the expert observes the complete sequence and assigns labels indicating whether it corresponds to LCA, RCA, or LV.

### Neural network architectures and training for feature extraction and classification

The dataset is consistently partitioned across all three models, ensuring uniform data distribution. We utilize 275 sequences in total for training and validation. 90% of the data is allocated for training and the remaining 10% for validation (247 and 28 sequences, respectively). To evaluate model robustness, an independent test set comprising 146 sequences (63 LCA, 38 RCA, and 45 LV) from a distinct distribution is employed.

#### Network architecture and training protocol for image data

**Fig. 13:**
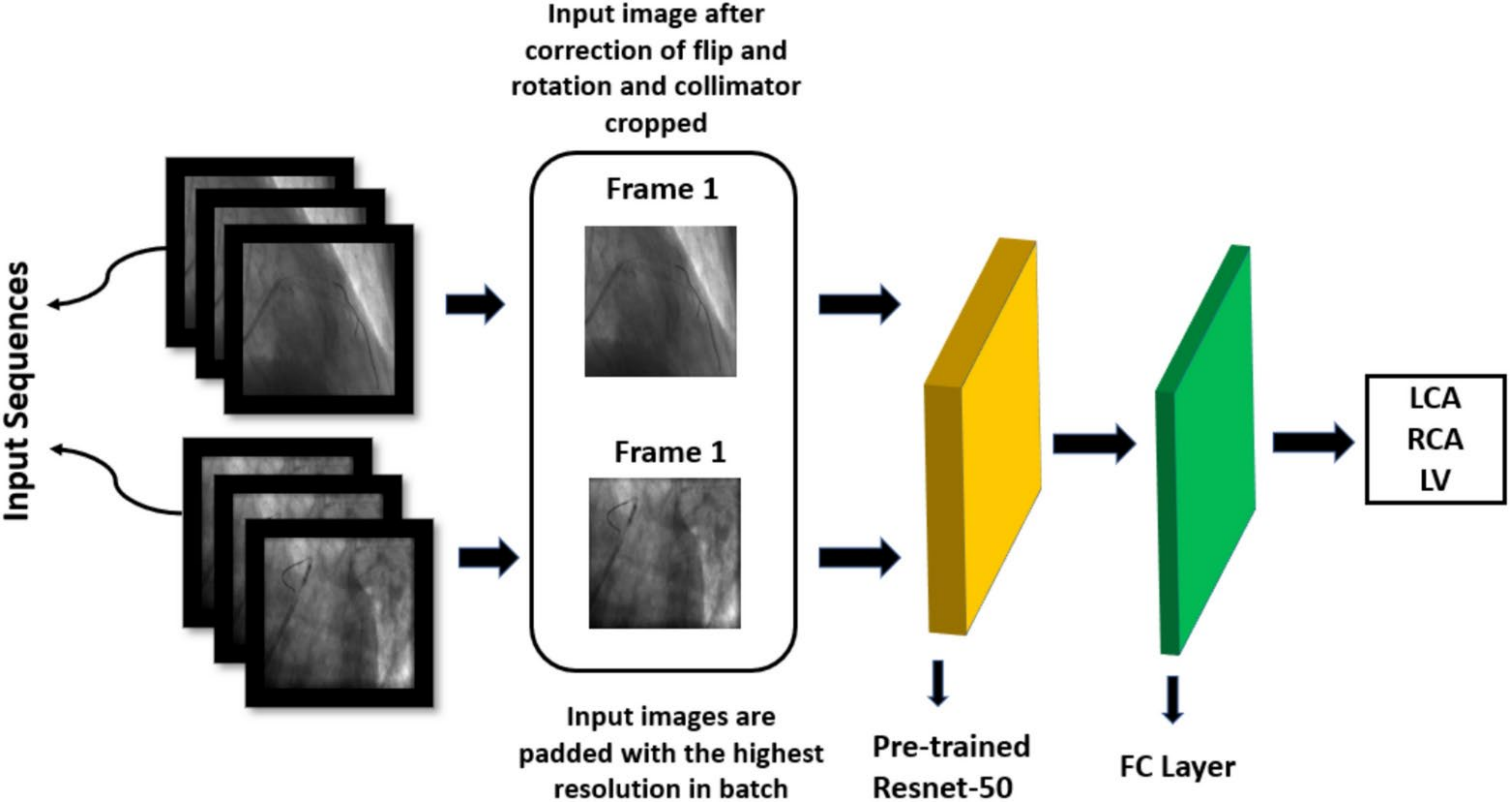
Proposed method: The input data includes the angiography sequences. The first frame of the sequence is considered and pre-processed to remove the collimator region and correct for flip and rotation. This image is provided as input to the Resnet-50 for feature extraction and then to the fully connected layer for final classification.

The network architecture for training on image data includes a pre-trained Resnet-50^24^ which uses ImageNet weights^25^. The prepared images described in section 3.1.2 are pre-processed using the techniques mentioned in section 2.2 and provided as input to Resnet-50 for feature extraction, and the extracted features are then passed through a fully connected layer for final classification. Figure 13 presents the proposed network architecture. The pre-processing techniques include log transform, square root transform, and scatter correction. The network outputs if the presented image belongs to the LCA, RCA, or LV class. Due to the image’s high resolution and limited memory capability a batch size of 2 was used and since the batch size is low a learning rate of 1e-4 were used with Adam optimizer as hyperparameters for training the model. Since we are working on a multiclass classification problem, we utilize categorical cross-entropy loss and the Softmax activation function. Early stopping was used with a patience value of 15 while monitoring the validation loss to avoid overfitting. The model converged around the 12th epoch.

#### Network architecture and training protocol for angulation data

**Fig. 14:**
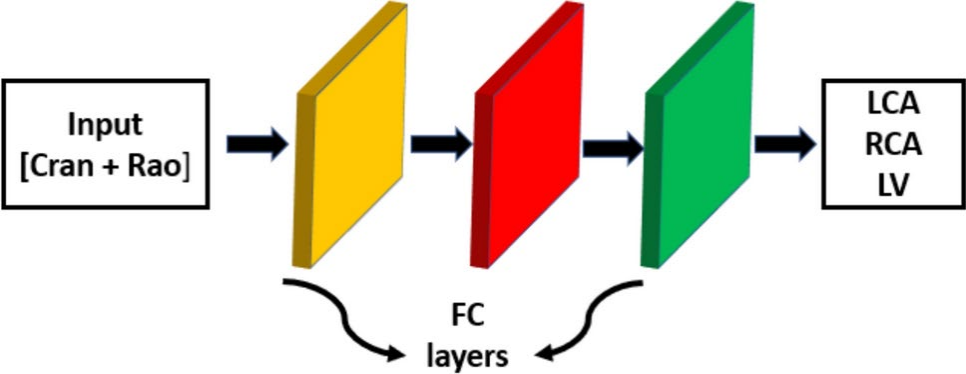
Proposed method: The input angulation data, which includes CRAN and RAO are passed through two fully connected layers for feature extraction and then finally through the last fully connected layer for classification.

A multi-layer perceptron MLP^26^ consisting of three layers is used for training this data. The first two layers are used for feature extraction, and the final layer is used for classification. Figure 14 presents the proposed network architecture. The model takes CRAN and RAO angles as input, without any normalization. It predicts whether the given angulation belongs to the LCA, RCA, or LV class. For the angulation data, we perform a 5-fold cross-validation with the same sample distribution as the Image data. This is performed because of the low number of samples for numeric data classification. For testing purposes, the angulation data from independent test data were used. Since the angulation data is a 1D vector, we set the batch size to 100 and a learning rate of 0.01 was used with Adam optimizer. As we work on a multiclass classification problem, we utilize categorical cross-entropy loss and Softmax activation function, similar to the X-ray image data model. We also implemented early stopping with a patience value of 25, while monitoring the validation loss. The model converged around the 139th epoch.

#### Multimodal network architecture and training protocol for multimodal data

**Fig. 15:**
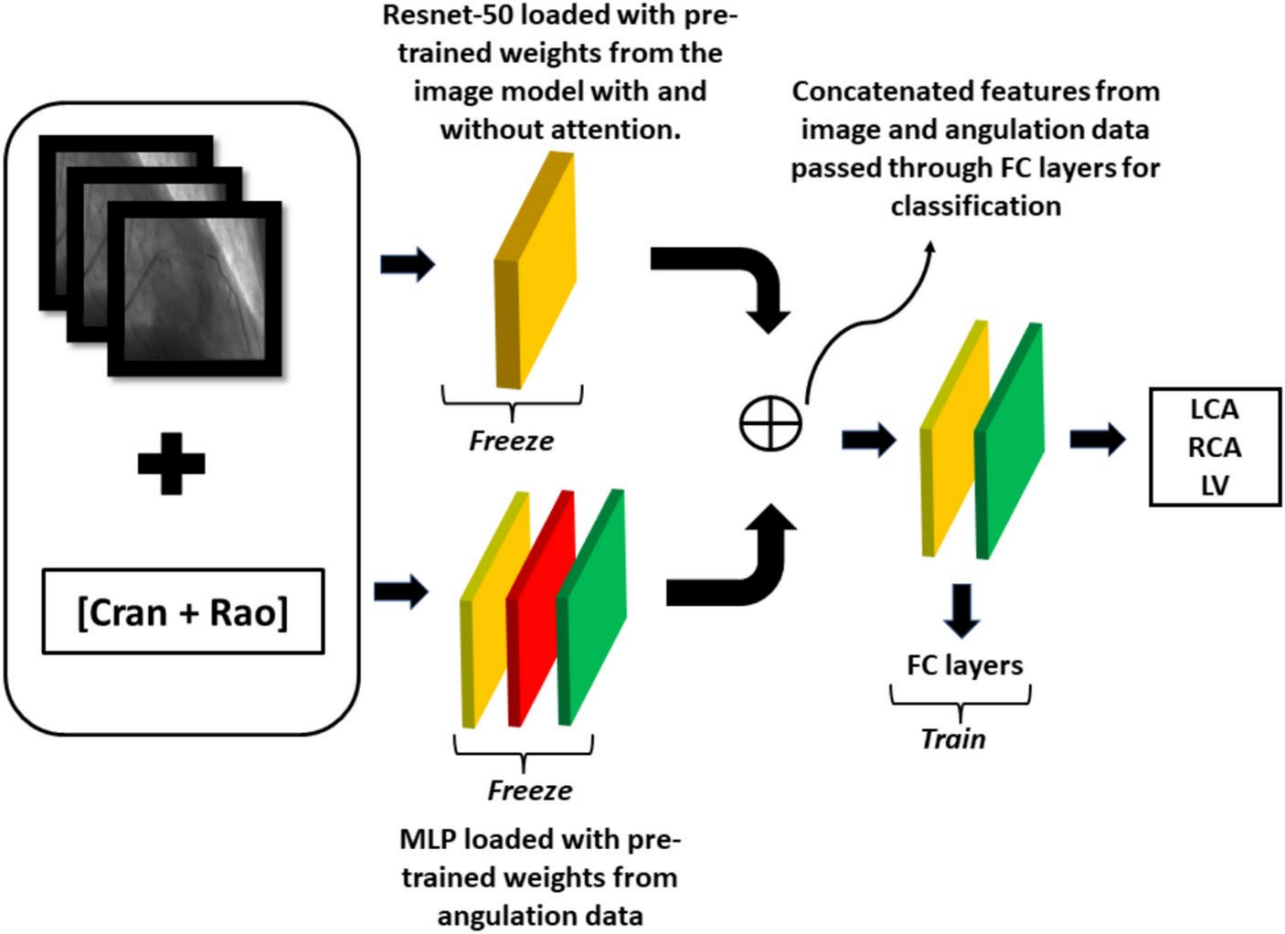
Proposed method: The input data for this model includes the image and angulation data combined. The image data is passed through a Resnet-50 model, which is loaded with pre-trained weights from the image model, and the layers are frozen. A similar method is applied to the MLP for handling angulation data. The output features from both models are concatenated and passed through a fully connected layer for final classification.

Figure 15 presents the proposed network architecture. It adopts transfer learning principles for anatomy classification^27^, utilizing a multimodal approach that combines a pre-trained ResNet-50 model for feature extraction of image data with a multi-layer perceptron (MLP) model for extracting angulation data features. Our model accepts two inputs, namely images and angulation data. A notable aspect of our approach is the use of fine-tuned models, which eliminates the need to train the network from scratch. We fine-tuned Resnet-50 with the weights of the image data model and the MLP with the weights of the angulation data model and froze all the layers with these weights. The input image data is processed through the Resnet-50 only for feature extraction and similarly, the angulation data is processed through MLP. The feature vectors generated by Resnet-50 and MLP are concatenated and then fed through a fully connected layer for classification between LCA, RCA, and LV. This fully connected layer is the only part of the network being trained, utilizing the combined features to determine the final output. The categorical cross-entropy loss function and Softmax activation function are used, similar to the other two models. Since we use both image and angulation data the memory requirement is higher, and we use a batch size of 2 and a learning rate of 1e-4 with Adam optimizer. We also implemented early stopping with a patience value of 25, while monitoring the validation loss. The model converged around the 111th epoch.

The content presented here is entirely self-authored; however, it has undergone paraphrasing with the assistance of ChatGPT to express the information in varied wording.

## Data Availability

The data used in this experiment were analyzed retrospectively. The data was obtained from a contract made between Siemens and the Hospital which states that the data can be used for any experiments and publication of Siemens. There were no changes made with respect to the treatment of the patient and only the acquired images were used for the experiment. The database is in the so-called raw format which makes it impossible to obtain any patient information. The study was performed according to the regulations and guidelines of Siemens and was approved by the Siemens quality committee “QR700000081”. The datasets analyzed during the current study are not publicly available due to data privacy restrictions of the contributing hospital but are available from the corresponding author upon reasonable request.

## Declarations

### Disclaimer

The information in this paper is based on research results that are not commercially available yet.
